# A Few Interesting Cases

**Published:** 1896-02

**Authors:** F. W. Maloney

**Affiliations:** Rochester, N. Y.; 332 West Avenue


### Clinical Report.

### A FEW INTERESTING CASES.

### Lupus Vulgaris—Syphilis—Erosion of Cervix Uteri—Endocervicitis with Prolapse of Bladder.

By F. W. MALONEY, M. D., Rochester, N. Y.

CASE I.—*Lupus Vulgaris.*—Miss F., aged 19, American; father died of pulmonary trouble; mother, a brother and a sister are living; came to me February 7, 1895. She had an ugly-looking nose, from which she had been suffering for six months, and had been taking medicine and local treatment. Crusts had accumulated to fully one-half inch in thickness on both sides of the nose, making it a disgusting object to look upon. She was anemic and run down in health. I began treatment by removing all the crusts and then, under cocaine anesthesia, with a sharp curette I scraped the ulcerated area quite thoroughly.

After bleeding had ceased, I applied pure lactic acid. The soft palate and upper surface of the throat contained several ulcers and these I also touched up with lactic acid. I ordered cod liver oil and a mixture with tincture chloride of iron, and bade her return on the next day. On the following day the diseased area was covered over with crusts, which were removed with some difficulty. I again applied lactic acid, and this I continued for ten days and at the end of that time there was no improvement whatever. There was an eroded appearance and the ulcers were spreading. I then used pure carbolic acid after another scraping, and applied unguentine. Followed this treatment every day for a week; no improvement. I then used nitrate of silver in stick form, and applied lanoline as an emollient, but abandoned this treatment after a week's trial. I then touched the ulcers with pure nitric acid for a few days; this seemed to freshen the surface, and the ulcers looked as though they were healing; but after a month had passed my patient was no better. I next turned to icthyol, and at the end of a week this seemed to take hold, there was quite a perceptible difference in the size of the ulcers, scabs were drying up, and at the end of two months of this treatment I discharged her as cured. But such was not the case; after six weeks, she returned and her nose looked as badly as before I began treatment. I scraped the parts again and applied icthyol, and kept it up every day for three weeks, but the ulcers would not heal. She was still taking the iron and cod liver oil. I applied aristol, sennine, and zinc ointment in their turn, and then went back to nitric acid, nitrate of silver, lactic acid and tincture perchloride of iron; another month passed by and no healing occurred. I was getting discouraged. I found nothing new in Burnet's System of the Nose and Throat, American Text-book of

Surgery, Reference Hand-book of the Medical Sciences and the current journal literature at my command. At the end of eight months she had the same ugly-looking nose as she had six months before. During the past few months my attention has been drawn to the brilliant results in the treatment of tuberculosis, diphtheria and other systemic diseases with protonuclein and nuclein solutions, as advocated by Prof. Aulde, of Philadelphia; Prof. Vaughan, of Michigan; Dr. Knapp, Dr. J. Mount Bleyer and others. As lupus is a tuberculous disease, I determined to make another effort for my patient, and procured an ounce of Messrs. Reed & Carnrick's special protonuclein powder. After again curetting the lupus area of the patient's nose, I dusted on the protonuclein from an ordinary powder blower, and did nothing more, but bid her to come next day. On the next visit the crusts were not so adherent and there seemed to be some improvement. After cleansing the surface I again dusted on the protonuclein powder. On the following visit there was a decided improvement, healthy granulations had sprung up, and a tendency to heal at the edges was apparent. On the third day the result was, indeed, surprising. No crusts formed; a dry surface remaining; the ulcers healthy and closing. I dusted the powder on the ulcers in the throat, and at the end of six days the nose had assumed a quite normal appearance, the tuberculous ridges smoothing out, all the ulcers completely healed, and those of the throat healing as well. This, to me, is a remarkable result in so short a time. Six months have now passed, and the patient says she feels better than she has in a long time.

CASE II.—*Tertiary Syphilis.*—Mrs. G., aged 34, married seven years; has no children; had sore throat and sore on lower lip before she was married. Was called to attend her on account of a large sore on the left side of the face. I found that the ulcers extended from the temporal region to the ramus of the lower jaw and including the left ear. There were several large holes eaten in the cheek, and the lower lobe of the ear had been eaten away. A thick, foul-smelling exudate covered the ulcers, which made the patient look as though she was suffering from one large rodent ulcer. The left eyelids were swollen, also the ear to twice its normal size. She said she experienced no pain to speak of, but that she could not see out of the left eye on account of the swollen condition. This state of affairs had been coming on about two weeks. I dusted the whole surface with the protonuclein powder and pinned on a clean piece of linen about the face. I ordered iodide of potash in xv. grain doses. When I saw her the next day, there seemed to be some improvement, as the swelling had gone down somewhat in the eyelids, and she said she took a few large pieces of the necrotic tissue out of the holes during the washing. I dusted the protonuclein powder well into the ulcers at each visit, and on the fourth day of the treatment there was a healthy granular appearance. The swelling in the eyelids and in the ear had subsided. I am satisfied that the potash salt alone could not bring about this result in so short a time. At the end of ten days there were no ulcers left ; the holes had filled in with healthy granulations, and these were even with the skin of the face. On the seventeenth day I discharged her cured. In this case the protonuclein seemed to act as a direct antitoxic and antiseptic, as well as a food to the tissues.

CASE III.—*Granular erosion of the cervix.*—Mrs. K., aged 32, mother of four children ; in second last confinement there was a torn cervix, which was not attended to at the time, or since. She came to me complaining of falling of the womb, backache and inability to do her housework. On examination, I found the uterus low in the pelvis. The cervix had been torn on the right side and about the external os was a severe state of granular process. The tear in cervix was small and as the patient was nursing her last baby I did not deem it advisable to operate. I dusted the protonuclein powder well into the erosions and applied a wool tampon, soaked in glycerine. Repeated the treatment twice a week. In two weeks the cervix had a healthy appearance and the granulations nearly all gone. In four weeks she felt well and cured.

CASE IV.—*Endocervicitis and complete prolapse of the bladder, ten days before confinement.*—Mrs. K., aged 30 ; had two children ; with the last one version was performed. Was called hurriedly, as she was about to be confined for the third time. I found her suffering a great deal of pain about the bladder. She said she had to urinate every ten minutes. On examination, I found a partial prolapse of the bladder. She had no real labor pains and after antiseptic precautions I pushed the bladder back with my hand. I found the cervix inflamed, some discharge, and the vaginal walls flabby. After a vaginal douche, I used a wool tampon saturated in glycerine, covered with the protonuclein powder and packed well against the cervix. I renewed the tampon every day for a week, when the patient said she felt well enough to get out of bed until her time should came. This I permitted her to do and three days after regular labor pains came on, when I delivered a full-term male child. I followed up the tampons and protonuclein powder for several days after and she is now feeling very well. I have since used the protonuclein powder on an injury to the vaginal wall after forceps delivery and did not have a particle of temperature.

Protonuclein seems to be nature's tissue builder and a direct antitoxic. It has to deal with the important function of cell life. Dr. Knapp says :

That it is sufficient to know that the cell is the seat of all the functions of the human body—nutritive, secretory, excretory and correlative—and that in health and disease we are concerned with the cell and not with the organism as a whole ; that the vital processes take place in the cell, and that the equilibrium between anabolism and katabolism, repair and waste, may be taken as a definition of health ; that certain physiological functions of the cells—chemotaxis, phagocytosis, cell proliferation and defensive proteids are the functions concerned ; immunity, vital resistance and the cure of disease.

That protonuclein increases the number of white cells has been clearly demonstrated by not a few investigators, and with this increase of white cells (leucocytosis) defensive action is produced, which becomes readily apparent in the treatment of disease.

332 West Avenue.

